# Psychological effects of project-based learning in participants receiving clinical oncology teaching: A protocol of systematic review of randomized controlled trials

**DOI:** 10.1097/MD.0000000000018514

**Published:** 2020-01-10

**Authors:** Ling-Qin Song, Jun-Li Han, Di Liu, Zhi-Jun Dai, Shu-Qun Zhang, Thomas Braun

**Affiliations:** a Department of Oncology; b Department of Critical Care Medicine, The Second Affiliated Hospital of Xi’an Jiaotong University (Xibei Hospital), Xi’an, China; c Medical School, University of Edinburgh, Teviot Place, Edinburgh, UK.

**Keywords:** effects, oncology medicine, project-based learning, randomized controlled trial

## Abstract

**Background:**

This study will assess the effects of the project-based learning (PBL) for participants undergoing clinical oncology teaching (COT).

**Methods:**

A systematic and comprehensive literature records will be identified from the electronic databases of PUBMED, EMBASE, Cochrane Library, Web of Science, Springer, Chinese Biomedical Literature Database, and China National Knowledge Infrastructure. All electronic databases will be searched from their inceptions up to the present. Any relevant randomized controlled trials on the effects of PBL in participants receiving COT will be considered for inclusion. Study quality will be assessed using the Cochrane risk of bias tool. RevMan 5.3 software will be utilized for statistical analysis.

**Results:**

This study will assess the effects of PBL in participants receiving COT through assessing the primary outcomes of psychological disorders, student satisfaction, and student feedback, and secondary outcomes of examination scores, excellence rates, course examination pass rates, and clinical knowledge or skills.

**Conclusion:**

The findings of this study will summarize the latest evidence on the effects of PBL in participants receiving in COT.

**PROSPERO registration number:**

PROSPERO CRD42019150433.

## 1 Introduction

Project-based learning (PBL) motivates participants to connect with knowledge and practical research experience, especially for the participants receiving clinical oncology teaching (COT).^[1–5]^ It has been reported that such method can help against the dissatisfaction with the traditional method of lecture-based learning.^[6–11]^ In addition, it also involves acquiring a deeper knowledge, as well as other skills, such as teamwork, cooperation, and make decisions.^[12–20]^ Previous studies have reported that PBL can significantly benefit for participants undergoing COT.^[21–32]^ However, its results are still inconsistent. Furthermore, no study has specifically focused on this issue. Therefore, this study will systematically and comprehensively assess the effect of PBL in participants receiving COT.

## 2 Methods

### 2.1 Study registration

This study has been funded through a protocol registry in the PROSPERO with CRD42019150433. It follows the Cochrane Handbook for Systematic Reviews of Interventions and the preferred reporting items for systematic reviews and meta-analysis protocol statement guidelines.^[33]^

### 2.2 Eligibility criteria for included studies

#### 2.2.1 Study types

We will include randomized controlled trials (RCTs) that assess the effects of PBL in participants receiving COT. However, we will exclude non-RCTs.

#### 2.2.2 Participant types

All college or university students who receive COT will be included with no restrictions of country, race, and sex.

#### 2.2.3 Intervention types

In the experimental group, all participants must receive PBL.

In the control group, all participants can receive any teaching methods, except the PBL.

#### 2.2.4 Outcome types

The primary outcomes are psychological disorders, as measured by any psychological assessment scales; student satisfaction, as measured by any relevant surveys or scores; and student feedback, as assessed by any student feedback Questionnaires.

The secondary outcomes consist of examination scores, excellence rates, course examination pass rates, and clinical knowledge or skills, as measured using any instruments.

### 2.3 Search strategy

We will systematically and comprehensively search the following electronic databases of PUBMED, EMBASE, Cochrane Library, Web of Science, Springer, Chinese Biomedical Literature Database, and China National Knowledge Infrastructure. We will search all electronic databases without limitations of language and publication status from their inceptions up to the present. The following search terms will be used: problem-based learning, PBL, internal medicine, cancer, education, randomized controlled trials, blind, concealment, control, and comparator. Any relevant studies on the effects of PBL in COT will be included. We will present detailed search strategy of PUBMED in Table 1. We will adapt similar search strategy for other electronic databases. We will also search for conference proceedings, and reference lists of related reviews.

**Table 1:**
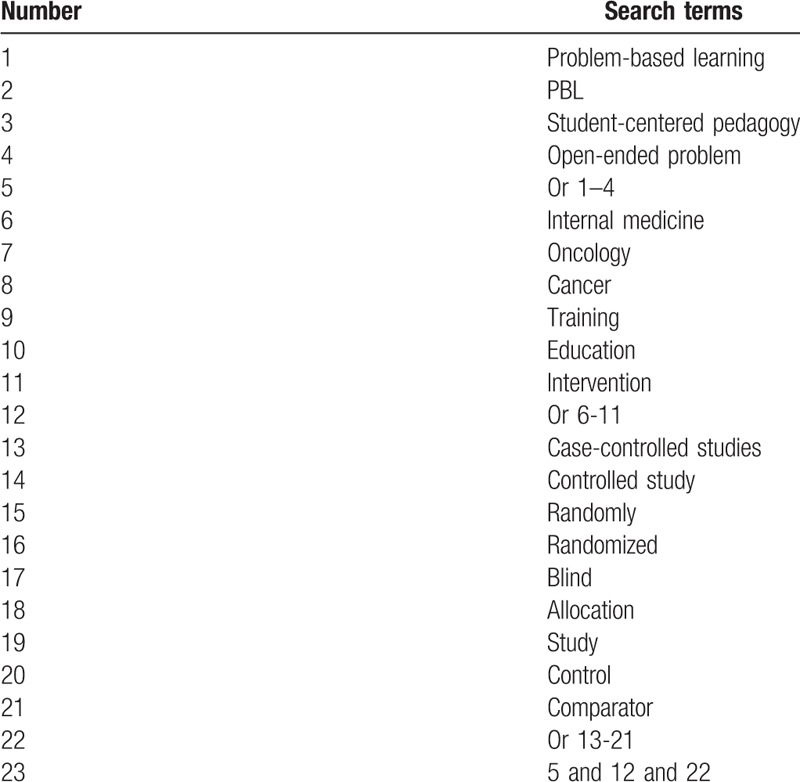
Search strategy for PUBMED database.

### 2.4 Data collection

#### 2.4.1 Study selection

Two independent authors will identify and review relevant studies based on the previous defined eligible criteria. Any disagreements regarding the study selection between 2 authors will be solved by a third author involved. First, they will check titles and abstracts for all searched literatures and all irrelevant and duplicated studies will be excluded. Then, we will read full-texts of all remaining studies to further judge if they meet all inclusion criteria. We will record all excluded studies with reasons. We will show the process of study selection in the flowchart.

#### 2.4.2 Data extraction

Two independent authors will carry out data extraction using standard designed data extraction sheet. A third author will be invited to solve any disagreements occurred between 2 authors. The extracted information is as follows: title, first author, time of publication, location, course name, study setting, participant characteristics, sample, study methods, intervention details, controls, outcomes, and other associated information. If any insufficient or missing information occurs, we will contact original authors to request it.

### 2.5 Risk of bias assessment

Two authors will independently evaluate the risk of bias assessment for each included study using the Cochrane Risk of Bias Tool. We will assess it on 7 levels, and each one is divided as low, unclear, and high risk of bias. If some different opinions exist between 2 authors, a third author will help to settle down them by discussion.

### 2.6 Statistical analysis

We will use RevMan 5.3 software for statistical analysis. We will calculate continuous data with mean difference or standardized mean difference and 95% confidence intervals (CIs), and will express dichotomous data as risk ratio and 95% CIs based on the availability of data from included studies. We will use *I*^2^ test to check heterogeneity among included studies. *I*^2^ ≤ 50% means low heterogeneity, while *I*^2^ > 50% means high heterogeneity. If low heterogeneity is identified among studies, we will use a fixed-effect model to pool the data. Meanwhile, we will plan to conduct meta-analysis if more than 2 studies on the same interventions and outcomes are included. On the other hand, if high heterogeneity is found, we will use a random-effect model to synthesize the data. At the same time, we will perform subgroup analysis and meta-regression test to check any possible factors that may result in such high heterogeneity among included studies.

### 2.7 Additional analysis

We will perform subgroup analysis based on the different interventions, study quality, and outcomes. In addition, we will also carry out sensitivity analysis to check the stability and robustness of pooled outcomes by removing studies with high risk of bias.

### 2.8 Reporting bias

If it is possible, we will also check the reporting bias using the funnel plot^[34]^ when more than 10 eligible RCTs enter in this study.

### 2.9 Ethics and dissemination

This study will not need ethic approval, because all data is collected from the published studies. Its results are expected to be published at a peer-reviewed journal.

## 3 Discussion

The PBL is one of the most common educational innovations developed against the dissatisfaction with the traditional education method. Recently, it has been widely utilized for the participants receiving COT. Up to now, a variety of studies have explored the effects of PBL compared with other education methods with inconclusive or inconsistent results. Furthermore, no systematic review has been addressed to investigate this issue. Thus, this study will assess the effect of PBL on the participants receiving COT. The results of this study will provide helpful evidence for clinical teaching education and future studies.

## Author contributions

**Conceptualization:** Ling-Qin Song, Di Liu, Zhi-Jun Dai, Shu-Qun Zhang.

**Data curation:** Ling-Qin Song, Jun-Li Han, Shu-Qun Zhang.

**Formal analysis:** Ling-Qin Song, Jun-Li Han, Di Liu, Shu-Qun Zhang.

**Funding acquisition:** Ling-Qin Song, Zhi-Jun Dai.

**Investigation:** Ling-Qin Song.

**Methodology:** Jun-Li Han, Di Liu, Shu-Qun Zhang.

**Project administration:** Ling-Qin Song.

**Resources:** Jun-Li Han, Di Liu, Zhi-Jun Dai.

**Software:** Jun-Li Han, Di Liu, Zhi-Jun Dai, Shu-Qun Zhang.

**Supervision:** Ling-Qin Song.

**Validation:** Ling-Qin Song, Jun-Li Han, Di Liu, Zhi-Jun Dai.

**Visualization:** Ling-Qin Song, Shu-Qun Zhang.

**Writing – original draft:** Ling-Qin Song, Jun-Li Han, Di Liu, Zhi-Jun Dai, Shu-Qun Zhang.

**Writing – review and editing:** Ling-Qin Song, Jun-Li Han, Di Liu, Zhi-Jun Dai, Shu-Qun Zhang.
